# Editorial: Novel Molecular Targets and Treatments for Gastroesophageal Cancer

**DOI:** 10.3389/fonc.2022.888861

**Published:** 2022-05-13

**Authors:** Alfred King-yin Lam, Bin Li, Linhui Liang, Jianjun Xie, Wen Wen Xu

**Affiliations:** ^1^ School of Medicine & Dentistry and Menzies Health Institute Queensland, Griffith University, Gold Coast, QSL, Australia; ^2^ The Fifth Affiliated Hospital of Guangzhou Medical University, Guangzhou, China; ^3^ Fudan University Shanghai Cancer Center and Institutes of Biomedical Sciences, Shanghai Medical College, Fudan University, Shanghai, China; ^4^ Department of Biochemistry and Molecular Biology, Shantou University Medical College, Shantou, China; ^5^ Guangzhou Medical University, Guangzhou, China

**Keywords:** esophagus, stomach, gastroesophageal, adenocarcinoma, squamous cell carcinoma, genomics, target therapy, molecular markers

In this Research Topic, we collected 20 papers under the title of “Novel Molecular Targets and Treatments for gastroesophageal Cancer” (Das et al., Power et al., Heng et al., Islam et al., Wang et al., Li et al., Zhang et al., Zhang et al., Deng et al., Bai et al., Lv et al., Guo et al., Chen et al., Luan et al., Jiang et al., Jafarzadeh and Soltani, Jin et al., Fang et al., Wang et al., Chen et al.). Cancers of the oesophagus and stomach account for 8.7% of new cases and 13.2% of new deaths of all sites worldwide (1). In the World Health Organization (WHO) classification of tumours, oesophageal cancer has two major histological types, namely squamous cell carcinoma (SCC) and adenocarcinoma (2). SCC is mostly noted in the upper and middle oesophagus and occurs mainly in high incidence regions such as in China, whereas adenocarcinoma is mostly in the lower oesophagus and oesophagogastric junction and is mostly in low incidence regions of high income and excess body weight. Recently, datasets reporting carcinoma of the oesophagus have been developed by the International Collaboration on Cancer Reporting (ICCR) (3, 4) to standardize the pathological reporting of cancer which allow a better base for research and improvement of management.

Among the papers focused on oesophageal cancer in this Research Topic, Das et al. review the therapeutic strategies against cancer stem cells, whereas Power et al. analyse immunotherapy approaches for oesophageal carcinomas. These papers open new avenues for innovative treatment of this cancer. The other papers are original studies based on SCCs from China, a high incidence area. Of these, Heng et al. studied the mechanisms and roles of camptothecin (anticancer agent) in oesophageal SCC cells. Islam et al. characterized the clinicopathological roles of molecular deregulation of *Endothelial PAS domain-containing protein 1 (EPAS1)* (code for an angiogenic factor) in 80 Hong Kong patients with oesophageal SCCs. In addition, the expression profiles of microRNAs could be useful as prognostic, and predictive biomarkers in oesophageal carcinomas (5). Wang et al. highlight the potential molecular target roles of miR-17-5p and miR-443 in the treatment of oesophageal SCC.

Spindle cell SCC is an uncommon subtype of SCC (2, 6). Li et al. analysed one of the largest series (n=43) of this cancer subtype in Southern China and developed a risk stratification and personalized management model. In the same centre, Zhang et al. analysed the ABO blood type in blood samples from 2179 patients with oesophageal carcinomas revealing that blood types had independent prognostic roles. Lastly, Zhang et al. from Shanghai reported the tumour-suppressive effect of Chinese herbal monomer, fangchinoline on oesophageal SCC cells.

Gastric cancer, predominately adenocarcinoma, is more common than oesophageal cancer, ranking fifth for incidence and fourth for mortality globally (1). Dataset reporting of carcinoma of the stomach has also been developed by ICCR (7) to standardize the pathological reporting of gastric carcinoma. In this area, Deng et al. reviewed the potential clinical value of tetraspanins in the management of gastric carcinoma. In addition, Bai et al. reviewed the advances and markers of immunotherapy in the treatment of patients with gastric adenocarcinoma and oesophagogastric adenocarcinoma. Lv et al. from China studied the expression of programmed death-ligand 1(PDL-1; predictor for immunotherapy), *HER-2* (*human epidermal growth factor receptor 2*; predictor for anti-HER 2-antibody therapy), immune microenvironment, and clinical features in 120 gastric adenocarcinomas. They noted that *HER-2* status could predict the efficacy of immune checkpoint inhibitors and *HER-2* status combined with PD-L1 level could predict the prognosis of patients tithe gastric carcinomas.

At the DNA level, Guo et al. analysed blood samples from 640 gastric adenocarcinomas from Chinese patients as well as gastric carcinoma cell lines and showed that *tumour necrosis factor alpha-induced protein 2* (*TNFAIP2*) polymorphism (rs8126 TC genotype) had a high risk of gastric carcinoma in male, elderly patients who are Helicobacter pylori-negative, non-smoking, and non-drinking individuals.

Gene expressions were studied in gastric carcinomas to investigate mechanistic pathways as well as their potential for target therapies. Chen et al. reported the expression of the transcription factor regulation gene, *PLXNC1* (*transcriptional factor plexin C1*) in 111 gastric adenocarcinomas from Chinese patients and gastric carcinoma cell lines. The results showed that *PLXNC1* plays an oncogenic role in gastric adenocarcinoma and could act as a therapeutic target. Luan et al. studied the expression of the TOR signalling pathway regulator (TIPRL) in 230 gastric carcinomas from Chinese patients, revealing that it suppresses cell migration and invasion by regulating the AMPK/mTOR signalling pathway in cancer. In addition, in 74 Chinese patients with gastric carcinoma and cancer cells, Jiang et al. showed that expression of fibronectin type III domain containing 1 (FNDC1) promotes the invasiveness of gastric cancer *via* the Wnt/β-catenin signalling pathway and correlates with peritoneal metastasis and prognosis.

Non-coding RNAs may include microRNAs, long noncoding RNAs (IncRNAs), and circular RNAs (cirRNAs) (8). Jafarzadeh and Soltanil from Iran demonstrated that InCRNA LOC400043 inhibits gastric cancer progression by regulating the Wnt signalling pathway in 15 gastric carcinomas and cell lines. In addition, Jin et al. demonstrated in 31 cases of gastric carcinomas from China and cancer cell lines that cirRNA promotes metastases under a long-term hypoxic microenvironment.

Proteins in carcinoma could alter tumour microenvironments such as matrix and cancer cell adhesions. In this aspect, Fang et al. studied the junctional adhesion molecular-like protein in 63 gastric carcinomas from Chinese patients and noted that it promotes tumour progression and metastases *via* the p38 signalling pathway. Wang et al. showed that a high level of legumain, with critical roles in extracellular matrix degradation and modelling, was associated with worse prognosis and peritoneal metastases in 139 Chinese patients with gastric carcinoma. Furthermore, Chen et al. studied the expression of myeloid differentiation factor 88 (MyD88), an adaptor molecule in Toll-like signalling pathway recognizing Helicobacter pylori, in 102 proximal gastric adenocarcinomas from Chinese patients by immunohistochemistry. MyD88 expression correlates with tumour grade and NF-kB p105/p50 expression.

To conclude, the papers in this Research Topic summarize current and novel molecular targets and treatments for oesophageal cancer and gastric cancer. This will enrich our understanding of pathogenesis and treatment possibilities, leading to the potential improvement of clinical outcomes of cancer.

## Author Contributions

AL conceptualized, designed, and wrote the editorial. All the authors contributed and approved the submitted version.

## Conflict of Interest

The authors declare that the research was conducted in the absence of any commercial or financial relationships that could be construed as a potential conflict of interest.

## Publisher’s Note

All claims expressed in this article are solely those of the authors and do not necessarily represent those of their affiliated organizations, or those of the publisher, the editors and the reviewers. Any product that may be evaluated in this article, or claim that may be made by its manufacturer, is not guaranteed or endorsed by the publisher.
